# Purvalanol A, Olomoucine II and Roscovitine Inhibit ABCB1 Transporter and Synergistically Potentiate Cytotoxic Effects of Daunorubicin In Vitro

**DOI:** 10.1371/journal.pone.0083467

**Published:** 2013-12-23

**Authors:** Daniela Cihalova, Jakub Hofman, Martina Ceckova, Frantisek Staud

**Affiliations:** Department of Pharmacology and Toxicology, Faculty of Pharmacy in Hradec Kralove, Charles University in Prague, Hradec Kralove, Czech Republic; Technische Universitaet Muenchen, Germany

## Abstract

Cyclin-dependent kinase inhibitors (CDKi) have high potential applicability in anticancer therapy, but various aspects of their pharmacokinetics, especially their interactions with drug efflux transporters, have not yet been evaluated in detail. Thus, we investigated interactions of five CDKi (purvalanol A, olomoucine II, roscovitine, flavopiridol and SNS-032) with the ABCB1 transporter. Four of the compounds inhibited efflux of two ABCB1 substrates, Hoechst 33342 and daunorubicin, in MDCKII-ABCB1 cells: Olomoucine II most strongly, followed by roscovitine, purvalanol A, and flavopiridol. SNS-032 inhibited ABCB1-mediated efflux of Hoechst 33342 but not daunorubicin. In addition, purvalanol A, SNS-032 and flavopiridol lowered the stimulated ATPase activity in ABCB1 membrane preparations, while olomoucine II and roscovitine not only inhibited the stimulated ATPase but also significantly activated the basal ABCB1 ATPase, suggesting that these two CDKi are ABCB1 substrates. We further revealed that the strongest ABCB1 inhibitors (purvalanol A, olomoucine II and roscovitine) synergistically potentiate the antiproliferative effect of daunorubicin, a commonly used anticancer drug and ABCB1 substrate, in MDCKII-ABCB1 cells as well as in human carcinoma HCT-8 and HepG2 cells. We suggest that this pronounced synergism is at least partly caused by (i) CDKi-mediated inhibition of ABCB1 transporter leading to increased intracellular retention of daunorubicin and (ii) native cytotoxic activity of the CDKi. Our results indicate that co-administration of the tested CDKi with anticancer drugs that are ABCB1 substrates may allow significant dose reduction in the treatment of ABCB1-expressing tumors.

## Introduction

Drug efflux transporters from the family of ATP-binding cassette (ABC) transport proteins, such as ABCB1 (P-glycoprotein, MDR1), ABCG2 (breast cancer resistance protein, BCRP), and ABCCs (multidrug resistance associated proteins, MRPs) mediate membrane transport of many endogenous substrates as well as xenobiotics. Abundantly expressed in tumor cells as well as physiological tissues, they play important roles in drug disposition, tissue protection and cancer resistance [1], [2], [3], thereby affecting pharmacokinetic/pharmacodynamic properties of many clinically used drugs [4]. The importance of identifying interactions of novel therapeutic agents with membrane drug transporters has recently been emphasized by regulatory agencies and many recommendations and decision trees for elucidating these interactions have been proposed [5], [6].

ABCB1 is the most extensively studied drug efflux transporter [7], [8]. Utilizing energy from ATP hydrolysis, it actively pumps structurally diverse compounds, including anticancer drugs, out of cells [9]. Two distinct drug binding and transport sites have been identified in ABCB1: the R- and H-sites, which bind rhodamine 123 and Hoechst 33342, respectively [10]. ABCB1 has become an attractive molecular target and inhibitors of this efflux transporter are being sought to increase the bioavailability of drugs after oral administration [11] or overcome drug resistance and sensitize cancer cells [12], [13].

Cyclin-dependent kinases (CDK) play important roles in the control of cell cycle progression and transcription. Thus, abnormalities in their regulation and expression can cause pathogenic changes resulting in various malignancies, and suppression of their activities by CDK inhibitors (CDKi) is a promising approach in cancer therapy [14], [15], [16], [17]. Several of these compounds are currently undergoing preclinical and clinical trials. Considerable attention has been devoted to their pharmacodynamic properties, but various pharmacokinetic aspects, especially their interactions with drug efflux transporters, have not yet been evaluated in detail.

In our previous studies we examined interactions of the prototypical purine CDKi olomoucine II and its derivative purvalanol A, with ABCG2, another important ABC transporter [18], [19]. The results revealed that these two compounds can inhibit ABCG2 in vitro and in situ and synergistically potentiate the antiproliferative effect of mitoxantrone in ABCG2-expressing cells. The aim of the study presented here was to characterize the inhibitory effect of several CDKi on the efflux activity of ABCB1. The selected set included olomoucine II, purvalanol A, roscovitine (another olomoucine II-derived drug), and the two most extensively studied CDKi that are currently undergoing clinical trials for treating various cancers: flavopiridol and SNS-032 [20], [21], [22]. To assess the ability of these compounds to inhibit ABCB1 transport activity, we examined their effects on the in vitro accumulation of Hoechst 33342 and daunorubicin (well established ABCB1 substrates that bind to the H- and R-sites of ABCB1, respectively) in MDCKII cells transduced with human ABCB1. We then further characterized these interactions by examining their ATPase activation and inhibition effects in ABCB1-overexpressing membrane vesicles. Moreover, as CDKi appear to be more clinically successful when co-administered with other cytotoxic agents [23], we hypothesized that interactive effects of the drugs on the ABCB1 transporter in tumor cells might intensify anticancer potency and strongly affect the outcome of treatments. To test this hypothesis, we applied each of the CDKi in combination with daunorubicin to ABCB1-expressing cells, both genetically modified and cancer-derived, to evaluate whether CDKi can synergistically potentiate daunorubicin’s cytotoxic effects.

## Materials and Methods

### Chemicals

Hoechst 33342 (HOE), daunorubicin (DNR), XTT sodium salt (XTT), phenazine methosulfate (PMS), purvalanol A and roscovitine (R-enantiomer) were purchased from Sigma Aldrich (St. Louis, MO, USA). ABCB1 inhibitor LY335979 (LY) was supplied by Toronto Research Chemicals (North York, ON, Canada). Olomoucin II was obtained from Merck (Darmstadt, Germany), flavopiridol and SNS-032 were purchased from SelleckChem (Houston, TX, USA). Cell culture reagents were supplied by Sigma Aldrich (St. Louis, MO, USA) and Gibco BRL Life Technologies (Rockville, MD, USA). An ABCB1 PREDEASY ATPase kit was purchased from Solvo Biotechnology (Szeged, Hungary).

### Cell lines

MDCKII cells transduced with the human ABCB1 gene (MDCKII-ABCB1) that stably express the ABCB1 transporter, and the parental MDCKII cell line, were obtained from Prof. Piet Borst and Dr. Alfred Schinkel (The Netherlands Cancer Institute, Amsterdam, The Netherlands). Both cell lines were grown in complete Dulbecco’s modified Eagle’s medium (DMEM) supplemented with 10% FBS. For in vitro drug combination studies we also used human ileocecal adenocarcinoma HCT-8 and human liver carcinoma HepG2 cell lines, both of which express ABCB1 [24]. The HCT-8 cells were purchased from the European Collection of Cell Cultures (HPA, Salisbury, Wiltshire, UK) and cultured in RPMI medium supplemented with 10% horse serum and 1 mM sodium pyruvate. The HepG2 cells, obtained from the American Type Culture Collection (LGC Promochem, Teddington, Middlesex, UK), were grown in minimal essential medium supplemented with 1 mM sodium pyruvate, 0.1 mM non-essential amino acids and 10% FBS. Cells from passages 5 to 30 were used in all in vitro studies. Dimethyl sulfoxide was applied as a CDKi solvent in concentrations not exceeding 0.5% (1% in ATPase assays).

### Absolute qRT-PCR quantification of ABCB1 transcripts in the cell lines

To evaluate and compare ABCB1 transcript levels in the cell lines used in this study, we used absolute real time RT-PCR quantification, as follows. Total RNA was isolated from each of the cell lines grown in culture flasks using TriReagent (Molecular Research Centre, Cincinnati, OH, USA) according to the manufacturer’s instructions. We measured the UV absorbance of the isolated RNA spectrophotometrically at 260 nm to determine its concentration and at 280 nm to check its purity from the 260/208 nm absorbance ratio using a NanoDrop spectrometer (Thermo Scientific, Wilmington, DE, USA). cDNA was prepared from 2 µg portions of the extracted total RNA with MMLV transcriptase using oligo(dT)18VN nucleotides and porcine RNase inhibitor (Tetro cDNA Synthesis Kit, Bioline, London, UK). We then amplified cDNA (from 40 ng of transcribed RNA) by real-time PCR using an iCycler (BioRad, Hercules, CA, USA) and 2× Probe Master Mix (Generi Biotech, Hradec Kralove, Czech Republic) in pre-designed PCR assays for ABCB1 (hABCB1_Q2, Generi Biotech, Hradec Kralove, Czech Republic). For absolute quantification, pCR plasmids (Generi Biotech, Hradec Kralove, Czech Republic) hosting subcloned PCR products of ABCB1 were used as PCR standards. Each sample and standard was amplified in triplicate, by incubation at 95°C for 3 min followed by 40 cycles of 95°C for 10 s and 60°C for 20 s. Standard curves were generated by preparing and amplifying seven decimal dilutions of the ABCB1 pCR plasmid, yielding copy numbers ranging from 2.5×10^1^ to 2.5×10^7^ copies per 20 µl reaction mixture. The resulting real-time amplification curves were analyzed, and threshold (Ct) values subtracted, using iCycler iQ 3.0 software (BioRad, Cincinnati, OH, USA). Excel software (Microsoft, Seattle, WA, USA) was used for all other calculations and the absolute number of cDNA copies in each sample was calculated from the generated calibration curves.

### HOE accumulation

To investigate the inhibitory activity of CDKi on ABCB1, the intracellular accumulation of HOE, a fluorescent ABCB1 substrate [25], was examined in both MDCKII-ABCB1 and MDCKII parent cell lines in the presence and absence of the tested compounds. The reduction in fluorescence intensity in ABCB1-transduced cells indicates the efflux activity of the ABCB1 transporter, its inhibition increases HOE accumulation and thus the intracellular fluorescence.

The assay was conducted as previously described [18] and optimized for application in ABCB1-expressing cells. Briefly, MDCKII-ABCB1 and MDCKII parent cells were seeded at 5×10^4^ cells per well on a 96-well culture plate and used for accumulation experiments after 24 h cultivation. The medium was removed and cells were washed twice with prewarmed phosphate buffered saline (PBS) at pH 7.4. They were then preincubated for 30 min (at 37°C in 5% CO_2_) with or without individual CDKi or LY (the potent ABCB1 inhibitor LY335979, [26]), each at eight selected concentrations. HOE was then added to 8 µM final concentration and fluorescence at 465 nm resulting from excitation at 350 nm was measured in 1 min intervals for 30 min using an Infinite 200 instrument (Tecan, Männedorf, Switzerland). The end-point fluorescence at t  =  30 min (after subtracting autofluorescence values of untreated cells) was used to calculate (IC_50_), the concentration of the tested CDKi providing 50% of its maximum inhibitory activity. For curve fitting and IC_50_ calculations, GraphPad Prism 5.04 (GraphPad Software Inc., La Jolla, CA, USA) was employed. As the tested CDKi did not reach the same maximum inhibitory levels (due to their cytotoxicity), the degree of ABCB1-mediated efflux inhibition by the individual compounds was assessed from their respective IC_50_ values.

### DNR accumulation

The accumulation of DNR, another fluorescent substrate of ABCB1 [27] that is known to bind to a site distinct from that of HOE [10], [28], was measured in an ABCB1-overexpressing cell line and compared to its accumulation in a control cell line lacking the transporter, using a previously published flow cytometry method [29], with slight modifications. Briefly, MDCKII-ABCB1 and MDCKII parent cells were seeded at 1.5×10^5^ cells per well on a 12-well plate 24 h before the experiment. The medium was removed and the cells were washed with prewarmed PBS. The cells were then preincubated in Opti-MEM, with or without CDKi or LY at 37°C in 5% CO_2_ for 30 min. DNR was then added to a final concentration of 2 µM and the cells were incubated under the same conditions for a further 60 min. Accumulation was stopped by cooling the samples on ice, removing the medium and washing twice with ice-cold PBS. The cells were then detached from the plates with 10× trypsin-EDTA, resuspended in PBS with 2% FBS and transferred to Eppendorf tubes, which were placed on ice until analysis. The intracellular DNR fluorescence of individual cells was analyzed using a C6 flow cytometer (Accuri, Ann Arbor, USA) with a 488 nm/585 nm excitation/emission filter and recorded as histograms. The median fluorescence (MF) intensity of 10,000 measured cells was used to compare the fluorescence resulting from each of the treatments. Viable cells (typically 60–80% of those measured) were gated based on forward and side scatter plots. The validity of the method was verified in control experiments using 7-aminoactinomycin D as a viability marker. The MF intensity of untreated cells was subtracted from fluorescence values obtained for all the measured samples. 1 µM LY was selected as a positive control because it can potently inhibit the ABCB1 efflux transporter.

To quantify the inhibitory effect of each tested compound on the ABCB1 transporter in the MDCKII-ABCB1 cell line, the ratio between the MF intensity with or without inhibitor was calculated and normalized to the effect in the parental MDCKII cell line according to the following equation [30]:
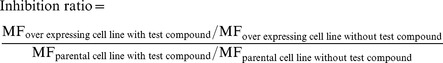



### ABCB1-ATPase assay

The drug efflux function of ABCB1 is linked to hydrolysis of ATP by ATPase, which is stimulated in the presence of ABCB1 substrates. In the activation assay, transported substrates can stimulate baseline vanadate-sensitive ATPase activity, whereas in the inhibition assay, which is carried out in the presence of a known activator of the transporter, inhibitors may reduce the maximal vanadate-sensitive ATPase activity. ATPase activity was measured by assessing the amount of phosphate liberated from ATP by the ABCB1 transporter using the PREDEASY ATPase kit for ABCB1 according to the manufacturer’s instructions. For this purpose, Sf9 cell membranes (4 µg protein per well) were mixed with each of the test compounds (singly) in solutions with concentrations ranging from 140 nM to 300 µM, then incubated at 37°C for 10 min in the presence or absence of 1.2 mM sodium orthovanadate. The reaction was started by adding 10 mM ATP solution (magnesium salt) to the reaction mixture, stopped 10 min later, and the absorbance at 590 nm was measured after a further 30 min incubation (GeniosPlus apparatus; Tecan, Männedorf, Switzerland). The ATPase activity in each sample was determined as the difference in liberated amounts of phosphate measured in the presence and absence of 1.2 mM sodium orthovanadate. Phosphate standards were prepared in each plate and verapamil served as a positive control for ABCB1 stimulation. The results are expressed as vanadate-sensitive ATPase activities.

### Cytotoxicity assay

1×10^4^ MDCKII-ABCB1, 1×10^4^ MDCKII parent, 2×10^4^ HCT-8, or 2×10^4^ HepG2 cells were grown in 96-well culture plates and incubated for 24 h. Individual CDKi diluted with growth medium were added to the exponentially growing cells and the resulting mixtures were incubated for 72 h at 37°C, 5% CO_2_. Cytotoxicity was then assessed using the XTT assay as follows: cells were incubated with 0.167 mg/mL XTT and 4 µM PMS in Opti-MEM for 2 h. The absorbance of the soluble formazan released was measured at 470 nm on a microplate reader (Tecan, Männedorf, Switzerland). The median effective antiproliferative concentrations (EC_50_) of the compounds were calculated using GraphPad Prism 5.04.

### Drug combinations

The combination index (CI) method of Chou-Talalay, based on the median-effect equation, was used to calculate combined drug effects. This approach offers quantitative definitions for additive, synergistic and antagonistic effects (CI values of 0.9–1.1, <0.9, and >1.1, respectively) [31]. Combination experiments were performed in a constant-ratio experimental design as recommended for the most efficient data analysis [32], and the generated data were used to quantify dose-reduction indices (DRI) for pairs of the tested drugs. DRI represents the fold-change of a focal effect when individual agents are used simultaneously relative to their separate effects, and their activity is synergistic if DRI > 1. The three ABCB1 inhibitors identified as most potent (purvalanol A, roscovitine and olomoucine II) in our accumulation experiments were combined with DNR, a commonly used anticancer drug and ABCB1 substrate. The XTT cytotoxicity assay was used to measure the cell viability in four cell lines (MDCKII-ABCB1, MDCKII parent, HCT-8 and HepG2) in the presence of the CDKi and DNR both singly and in combination, at constant concentration ratios, ranging from 0.1 to 1.5 multiples of their respective, predetermined EC_50_ values. The data acquired from these drug combination experiments were analyzed using CompuSyn ver. 3.0.1 software (ComboSyn Inc., Paramus, NJ, USA).

### Statistical analysis

Data are presented as means ± SD. Between-treatment differences, calculated using ANOVA or Student’s t test implemented in GraphPad Prism 5.04, are considered significant if *P*<0.05.

## Results

### Expression of ABCB1 mRNA in the cell lines

Expression of the gene encoding human ABCB1 transporter was quantified in all cell lines used in this study. Levels of ABCB1 transcripts were highest in the MDCKII-ABCB1 line: more than an order of magnitude higher than in HepG2 and HCT-8 cells. As expected, no transcripts of the human ABCB1 gene were detected in the parental MDCKII cell line (Table 1).

**Table 1:** Number of ABCB1 transcripts per µg of total RNA in each of the cell lines.

	MDCKII-parent	MDCKII-ABCB1	HCT-8	HepG2
*ABCB1* Transcripts (10^4^)	ND	3100±288^ a^	35.2±9.65	153±16.9

Presented data are means ± SD of three experiments performed in triplicate. ^a^ Significantly different from ABCB1 expression in HCT-8 and HepG2 cell line (*P*<0.001) as analyzed by ANOVA followed by Bonferronís test. ND, no transcripts detected.

### Effect of CDKi on ABCB1-mediated efflux of HOE from MDCKII-ABCB1 cells

**Figure 1:**
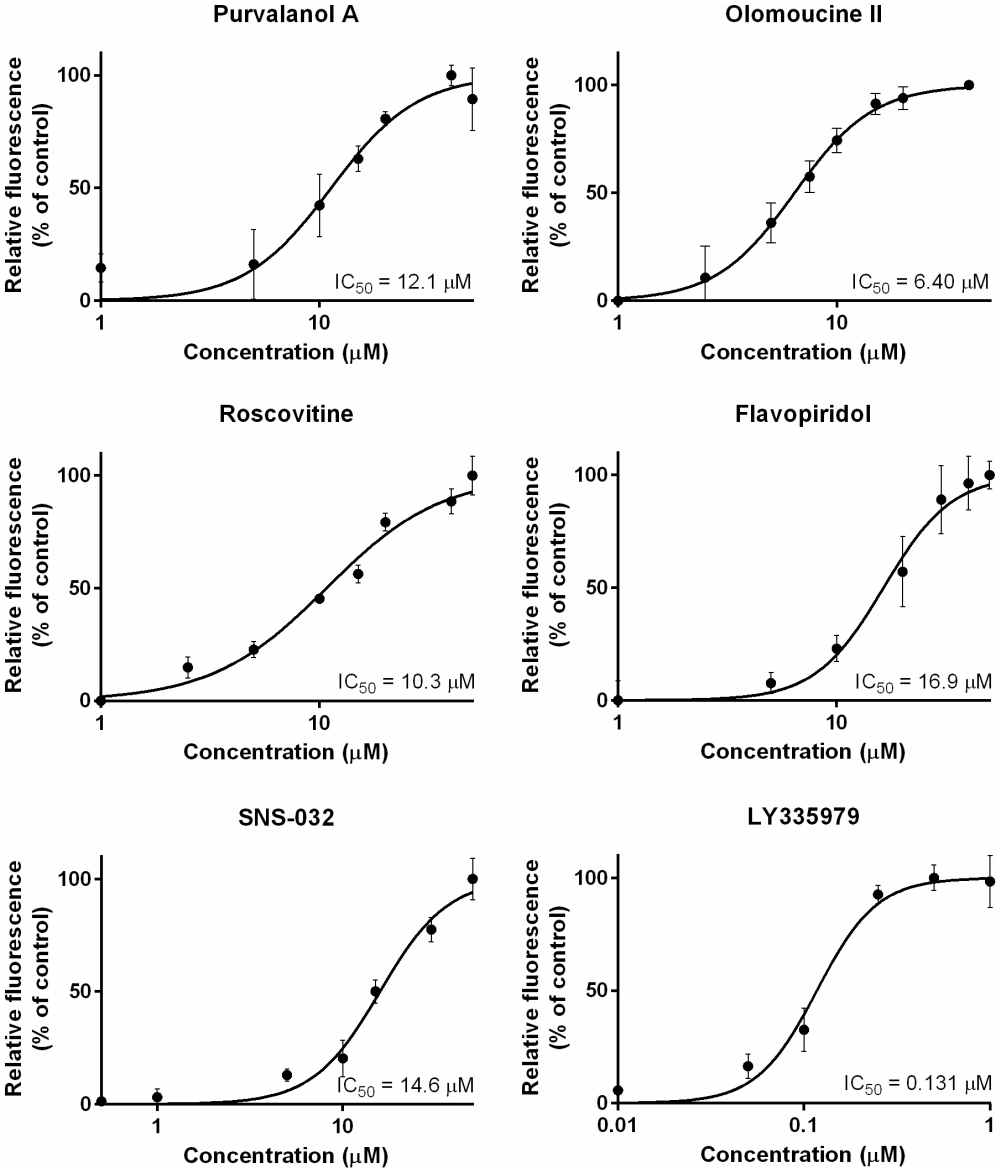
Effects of CDKi and the model ABCB1 inhibitor LY on ABCB1-mediated efflux of HOE in MDCKII-ABCB1 cells. 0% and 100% respectively indicate the fluorescence of unaffected control cells and the maximal fluorescence observed in assays with a particular CDKi. Presented data are means ± SD obtained from three independent experiments performed in triplicate.

**Figure 2:**
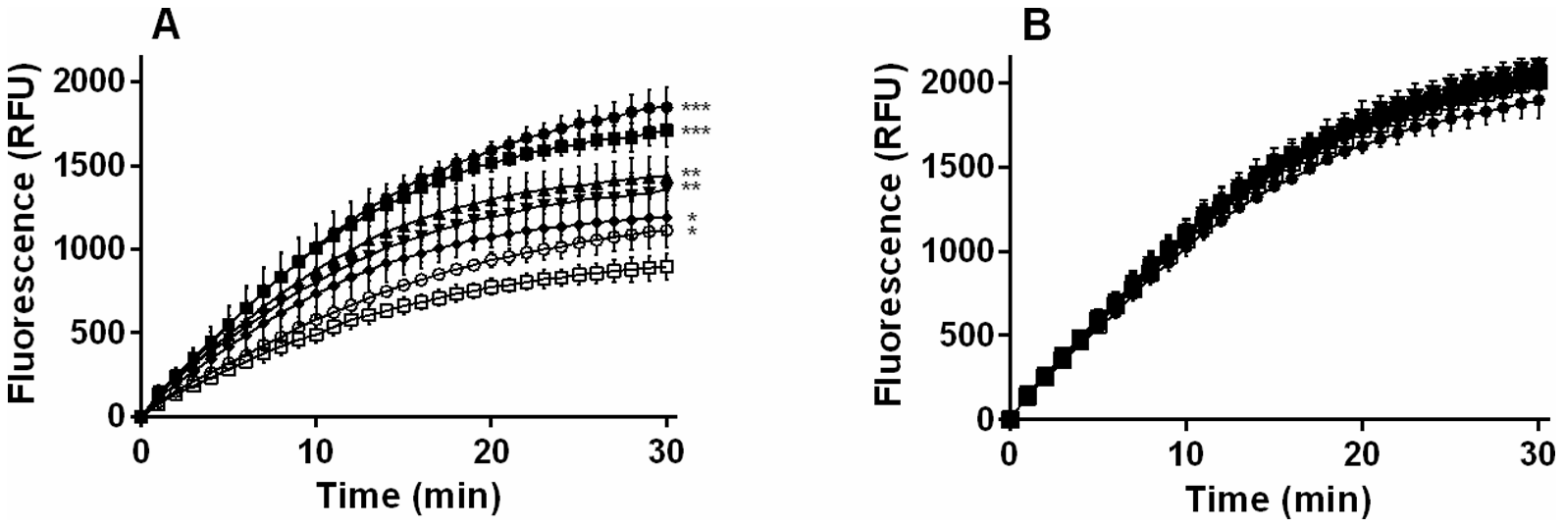
Time-dependent accumulation of HOE in MDCKII cells. MDCKII-ABCB1 (A) and MDCKII parent (B) cells were incubated in the absence (control □) or presence of purvalanol A (▪), roscovitine (▴), olomoucine II (▾) flavopiridol (⧫) or SNS-032 (○) at their respective IC_50_ concentrations. A potent ABCB1 inhibitor, LY (•), was used as a positive control for ABCB1 inhibition. Presented data are means ± SD obtained from three independent experiments performed in triplicate. The statistical significance (*P<0.05; **P<0.01; ***P<0.001) of differences in HOE levels detected in treated and control cells was determined using unpaired *t* tests.

All tested CDKi inhibited ABCB1-mediated efflux of HOE in MDCKII-ABCB1 cells (Fig. 1), with potency declining in the following order: olomoucine II > roscovitine > purvalanol A > SNS-032 > flavopiridol (IC_50_  =  6.4, 10.3, 12.1, 14.6 and 16.9 µM, respectively). However, all of the compounds were much less potent than the model ABCB1 inhibitor, LY (IC_50_, 0.131 µM). When the CDKi were applied at their respective IC_50_ concentrations, purvalanol A inhibited HOE efflux from MDCKII-ABCB1 cells most strongly (86% as strongly as LY) followed by roscovitine, olomoucine II, flavopiridol and SNS-032 (57%, 48%, 31% and just 23% as strongly as LY, respectively). The accumulation of HOE in the MDCKII parent cell line was unaffected by addition of the CDKi (Fig. 2).

### Effect of CDKi on ABCB1-mediated efflux of DNR from MDCKII-ABCB1 cells

Based on the results of the HOE efflux experiments, the CDKi were each applied at three concentrations (1, 10 and 20 µM) to investigate their effects on the ABCB1-mediated efflux of DNR. LY (1 µM) was applied as a positive control for ABCB1 inhibition. At CDKi concentrations above 20 µM and LY concentrations above 1 µM cells drifted out of the gates, thus the resulting data were not included in the analysis.

**Figure 3:**
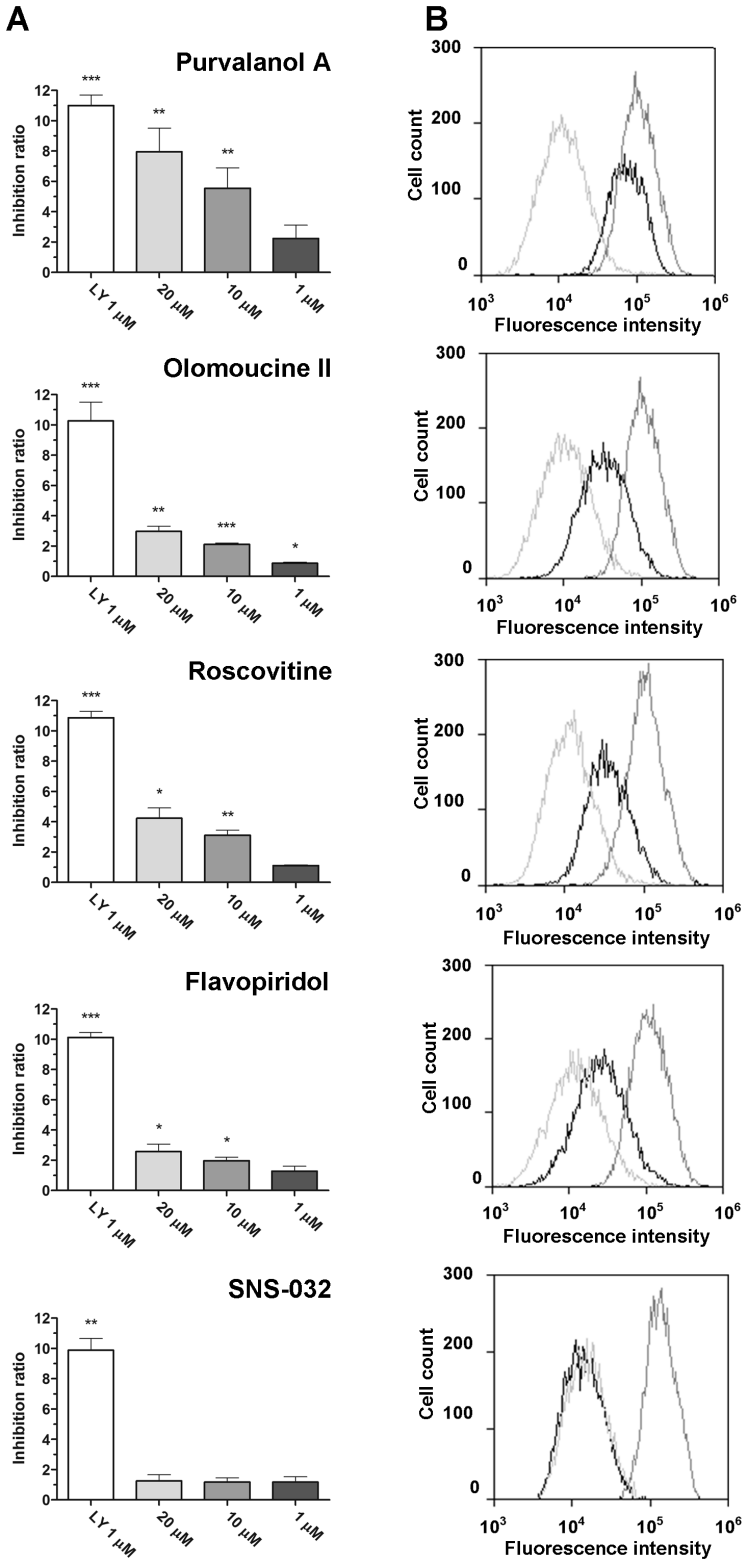
Effect of the tested CDKi on intracellular accumulation of DNR in MDCKII-ABCB1 cells. (A) Inhibition, expressed as the ratio between the median fluorescence with and without the indicated inhibitors during the efflux period, normalized to the effect in the parental cell line MDCKII (see *Materials and Methods* for details). LY (1 µM) was used as a model inhibitor. Presented data are means ± SD obtained from three independent experiments performed in duplicate. *P* values of differences between observed inhibition ratios and the null hypothetical value of 1 (no ABCB1 inhibition) were determined by unpaired two-tailed *t* tests: *P<0.05; **P<0.01; ***P<0.001. (B) Representative histograms of data obtained in assays with each compound at 20 µM: control with no inhibitor (light grey), tested compound (black), 1 µM LY (dark grey).

All of the tested CDKi inhibited DNR efflux dose-dependently across the applied range, 1 - 20 µM, but less strongly than LY (Fig. 3). At 1 µM they showed at most slight inhibitory activity, in accordance with the results of our HOE accumulation studies. However, at the highest concentration (20 µM), four CDKi exhibited significant (P<0.05) ABCB1 inhibition, declining in the order purvalanol A > roscovitine > olomoucine II > flavopiridol. In contrast to its observed inhibitory effect in HOE accumulation assays, SNS-032 did not inhibit DNR accumulation at any tested concentration.

### Effects of CDKi on ATPase activity in ABCB1-containing membrane preparations

**Figure 4:**
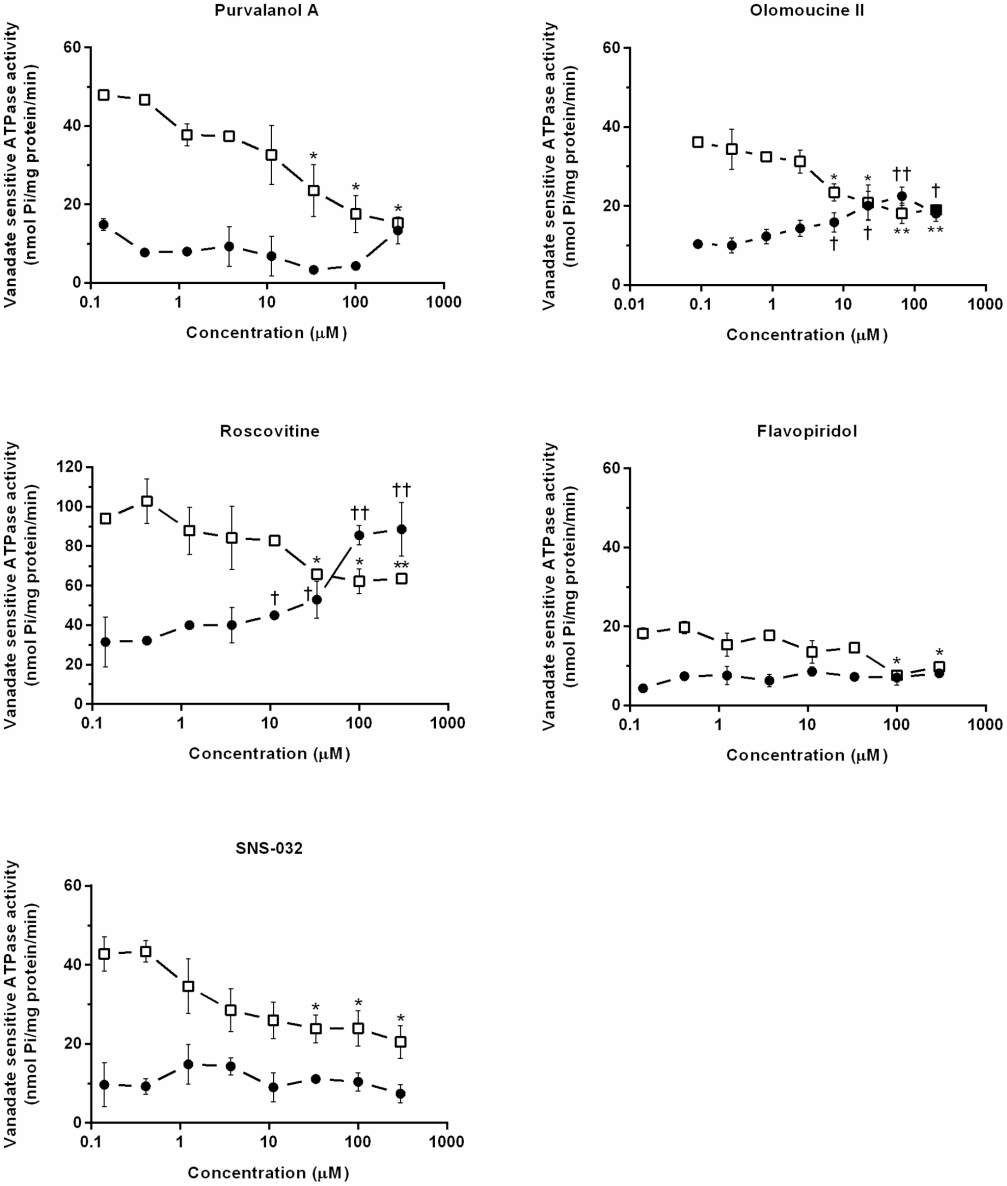
Effects of CDKi on the ATPase activity of ABCB1-Sf9 membrane preparations. Vanadate-sensitive ATPase activity in the presence of purvalanol A, olomoucine II, roscovitine, flavopiridol, or SNS-032 in activation (•) and inhibition (□) experiments. Presented data are means ± SD representative of at least two experiments performed in duplicate. The significance of differences linked to the absence and presence of CDKi in the basal activity of the transporter in activation assays (†*P*<0.05; ††*P*<0.01; †††*P*<0.001) and the activity of the activated transporter in inhibition assays (*P<0.05; **P<0.01; ***P<0.001) were determined using unpaired *t* tests.

To further characterize interactions of the CDKi with ABCB1, we tested their modulatory effects on vanadate-sensitive ATPase activity in isolated insect Sf9 cell membranes overexpressing human ABCB1. In the inhibition study, purvalanol A, olomoucine II and roscovitine considerably and dose-dependently reduced the verapamil-stimulated vanadate-sensitive ATPase activity of ABCB1 while flavopiridol and SNS-032 only slightly reduced it at the highest tested concentration. In the ATPase activation assay, roscovitine and olomoucine II (but not purvalanol A, flavopiridol or SNS-032) increased the baseline vanadate-sensitive ATPase activity of ABCB1 (Fig. 4).

### Determination of synergistic antiproliferative activity of CDKi and DNR in combination

**Figure 5:**
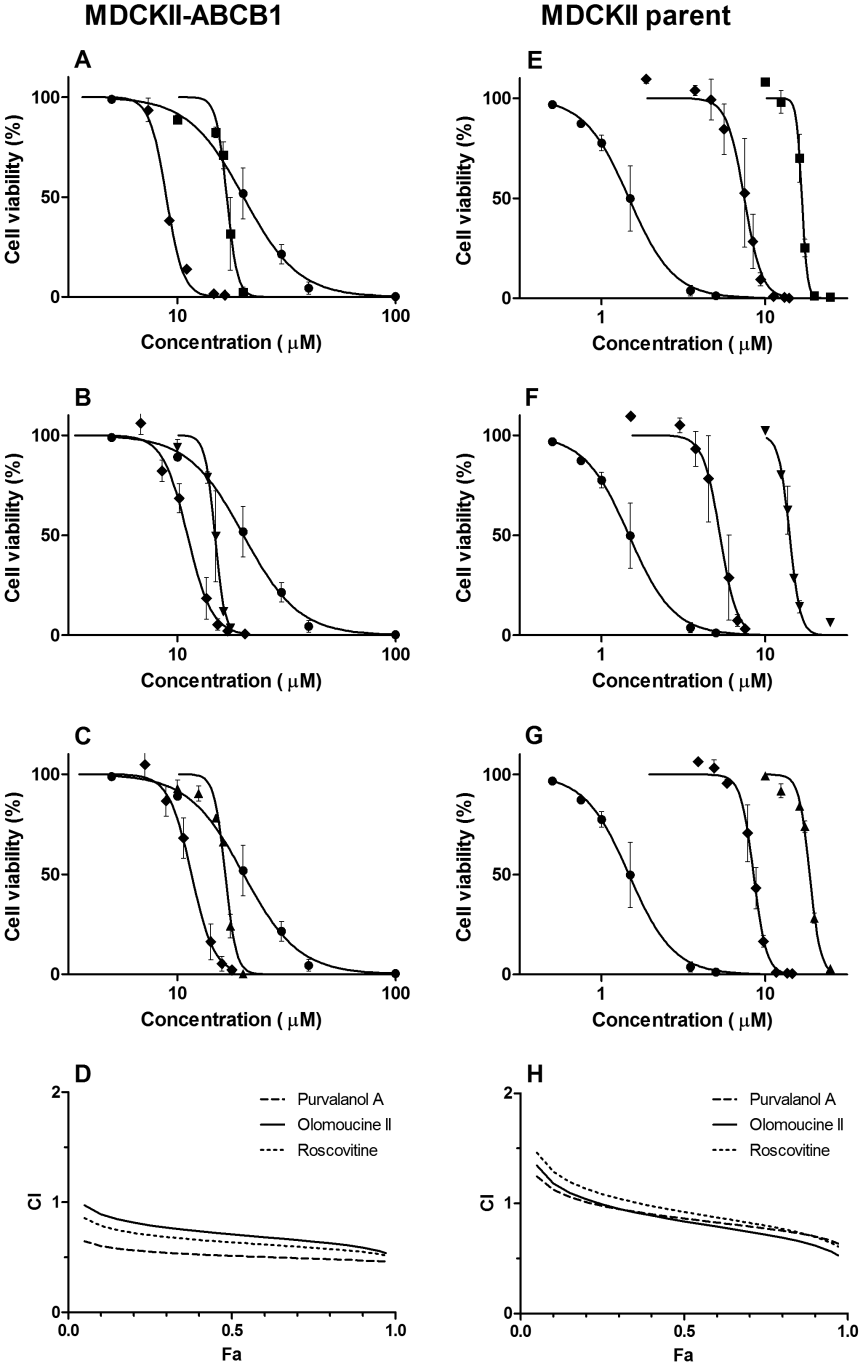
Cytotoxicity and combination experiments in MDCKII-ABCB1 and parental cell lines. Cytotoxic effect of (A, E) purvalanol A (▪), (B, F) olomoucine II (▾), (C, G) roscovitine (▴) or daunorubicin (•) and their combination (⧫) on MDCKII-ABCB1 or MDCKII parent cells. For combinations, concentrations corresponding to particular points in the graph are sums of concentrations of the individual drugs administered in fixed concentration ratios (Table 2), based on the ratio of their respective EC_50_ values. Presented data are means ± SD obtained from at least three independent experiments performed in triplicate. (D, H) The cytotoxic effect (combination index, CI, plot) of CDKi and daunorubicin combinations on MDCKII-ABCB1 or MDCKII parent cells, obtained using CompuSyn software. Fractional effects (Fa) were calculated from the cell viability values of individual compounds; Fa  =  0 means no antiproliferative effect, Fa  =  1 means 100% antiproliferative effect. CI  =  0.9–1.1 indicates additive effect, CI <0.9 synergism and CI > 1.1 antagonism.

To assess whether the tested CDKi can synergistically potentiate the effect of another concomitantly administered cytotoxic compound that is known to be an ABCB1 substrate, we employed the combination index method of Chou-Talalay. The CI values obtained from applications of purvalanol A, olomoucine II and roscovitine in combination with DNR to the MDCKII-ABCB1 cell line fell in the synergistic category of drug combination effects across almost the whole fraction of cells affected (Fa) range (Fig. 5A-D). In contrast, significantly weaker synergistic effects were observed in the MDCKII parent cell line, where combinations of purvalanol A, olomoucine II and roscovitine with DNR only displayed synergistic effects when the Fa exceeded 0.4, 0.4 and 0.45, respectively (Fig. 5E-H). The calculated DRI indicate that the presence of purvalanol A, olomoucine II or roscovitine allows 4.6-, 3.3- or 3.8-fold reductions in the DNR doses required to reach an Fa of 0.75 in MDCKII-ABCB1 cells (Table 2).

**Table 2:** Dose reduction index (DRI) values for drug combinations scheduled after 72 h of simultaneous treatment.

Cell line	Drugs		Concentration ratio	DRI at Fa					
				0.5		0.75		0.9	
	I	II	I∶II	I	II	I	II	I	II
MDCKII-ABCB1	DNR	Purvalanol A	1∶0.9	3.76±0.07	4.03±0.08	4.56±0.07	3.73±0.06	5.54±0.06	3.45±0.04
		Olomoucine II	1∶0.7	2.68±0.26	2.83±0.27	3.32±0.22	2.84±0.19	4.12±0.21	2.86±0.10
		Roscovitine	1∶0.8	3.06±0.03	3.20±0.03	3.80±0.03	3.08±0.02	4.72±0.05	2.96±0.03
MDCKII parent	DNR	Purvalanol A	1∶11.7	2.25±0.03	2.41±0.03	2.84±0.05	2.40±0.04	3.60±0.09	2.39±0.06
		Olomoucine II	1∶9.2	2.38±0.08	2.51±0.08	3.07±0.06	2.63±0.05	3.96±0.03	2.75±0.02
		Roscovitine	1∶12.3	ND	ND	2.74±0.06	2.29±0.05	3.57±0.09	2.37±0.06
HCT-8	DNR	Purvalanol A	1∶17.7	ND	ND	3.12±0.03	1.77±0.01	4.36±0.13	1.68±0.05
		Olomoucine II	1∶6.3	3.11±0.13	3.04±0.13	4.14 ±0.08	2.98±0.06	5.45±0.02	2.92±0.05
		Roscovitine	1∶17.0	2.63±0.02	2.49±0.02	3.96±0.05	2.29±0.03	5.96±0.17	2.11±0.06
HepG2	DNR	Purvalanol A	1∶43.0	ND	ND	ND	ND	4.05±0.08	1.68±0.03
		Olomoucine II	1∶20.5	ND	ND	2.55±0.03	2.08±0.03	4.68±0.09	2.38±0.04
		Roscovitine	1∶47.0	ND	ND	2.72±0.02	2.51±0.02	5.02±0.03	2.56±0.02

ND, not determined.

**Figure 6:**
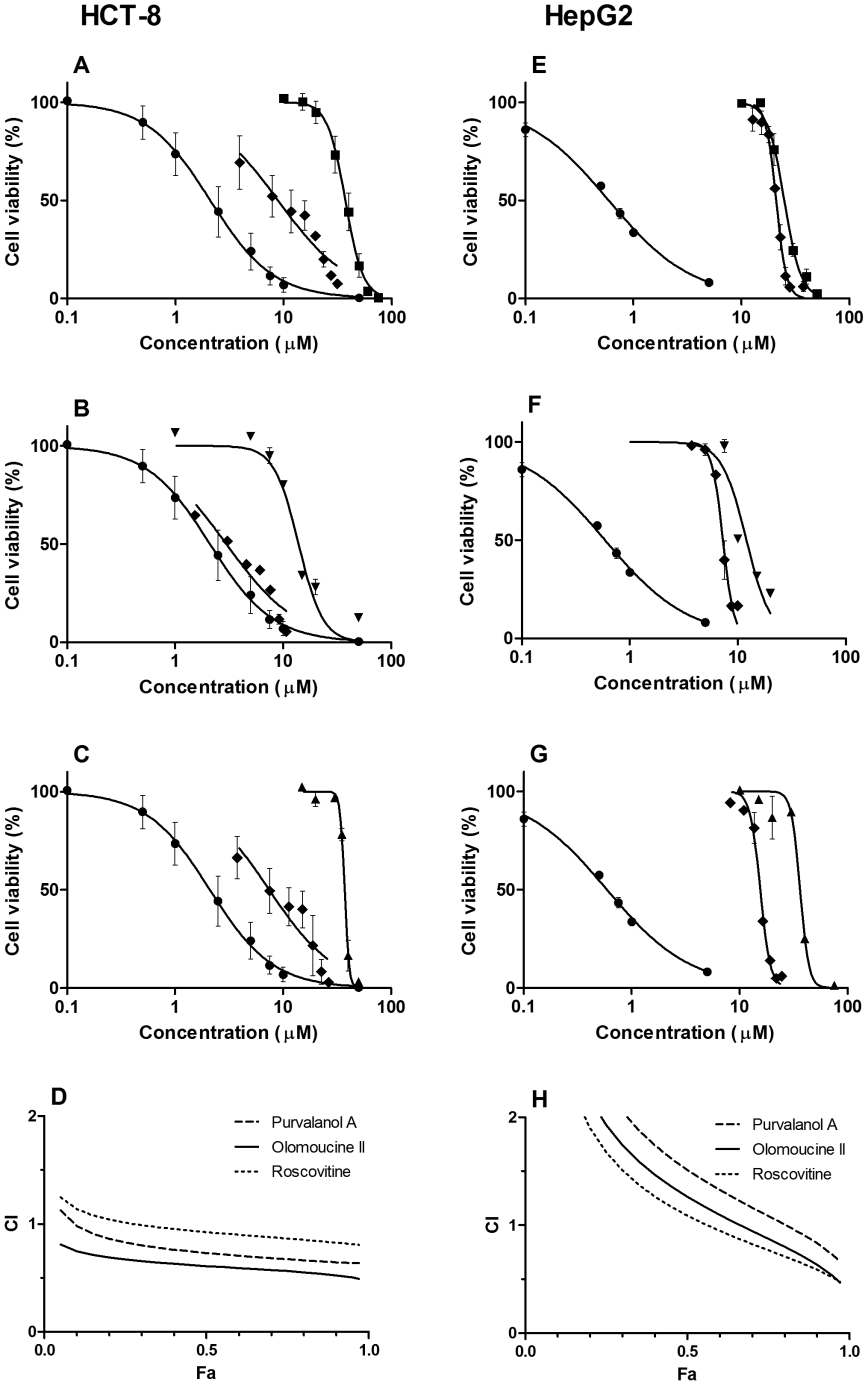
Cytotoxicity and combination experiments in HCT-8 and HepG2 cell lines. Cytotoxic effect of (A, E) purvalanol A (▪), (B, F) olomoucine II (▾), (C, G) roscovitine (▴) or daunorubicin (•) and their combination (⧫) on HCT-8 cells or HepG2. For combinations, concentrations corresponding to particular points in the graph are sums of concentrations of the individual drugs administered in fixed concentration ratios (Table 2), based on the ratio of their respective EC_50_ values. Presented data are means ± SD obtained from at least three independent experiments performed in triplicate. (D, H) The cytotoxic effect (combination index, CI, plot) of CDKi and daunorubicin combinations on MDCKII-ABCB1 or MDCKII parent cells, obtained using CompuSyn software. Fractional effects (Fa) were calculated from the cell viability values of individual compounds; Fa  =  0 means no antiproliferative effect, Fa  =  1 means 100% antiproliferative effect. CI  =  0.9–1.1 indicates additive effect, CI <0.9 synergism and CI >1.1 antagonism.

In the HCT-8 cell line, combinations of DNR with olomoucine II, roscovitine and purvalanol A showed synergistic cytotoxic effects at Fa >0.1, >0.3 and >0.75, respectively (Fig. 6A-D). Corresponding Fa values for synergism in the HepG2 cell line were 0.75, 0.65 and 0.9, respectively (Fig. 6E-H).

## Discussion

CDKi are a promising class of anticancer [15], [16] and antiviral [33], [34], [35] drugs. The cell cycle-related effects of these compounds have been intensively researched, but their interactions with drug efflux transporters have not been previously evaluated in detail. Thus, in the presented study we employed several experimental approaches to elucidate interactions of five CDKi (purvalanol A, olomoucine II, roscovitine, flavopiridol, and SNS-032) with the ABCB1 transporter in vitro.

Using accumulation assays in MDCKII-ABCB1 cells, we show that all tested CDKi inhibit the ABCB1 transporter. We demonstrate that olomoucine II, roscovitine, purvalanol A and flavopiridol can inhibit ABCB1-mediated efflux of both HOE and DNR, indicating that the drugs can interact with the H- as well as R-site of the ABCB1 transporter. In contrast, SNS-032 selectively inhibited ABCB1-mediated transport of HOE, but not DNR, suggesting that this compound interacts with efflux activity of the H-site, but not R-site, of ABCB1. Preferential affinity of substrates and inhibitors for either of the two ABCB1 binding sites is thus an important factor to consider when investigating and predicting ABCB1-mediated drug-drug interactions, as recently demonstrated by Wang et al. [36].

To further characterize the interactions of CDKi with ABCB1 we examined their effects on the activities of ATPase in Sf9 membranes overexpressing human ABCB1. All the tested substances decreased activation of ABCB1 ATPase, confirming that they interact with the ABCB1 transporter. Our results also provide the first indications that olomoucine II is an ABCB1 substrate and inhibitor, as well confirming previous observations that roscovitine has these characteristics [37]. In contrast, purvalanol A, flavopiridol and SNS-032 can be classified as non-substrates of ABCB1 as they did not affect the ATPase activity. Interestingly, flavopiridol has recently been shown to be transported by mouse Abcb1 [38], [39]. Similarly, higher levels of SNS-032 have been detected in brains of Abcb1 knockout mice than in wild type mice, suggesting that SNS-032 is a substrate of mouse P-glycoprotein [40]. We believe these discrepancies may be due to interspecies differences, in accordance with observations recently reviewed by Chu et al. [41].

In cancer treatment, drugs are frequently administered in various combinations to increase their therapeutic effects, reduce toxicity, and minimize the induction of drug resistance [32], [42]. Here we hypothesized that simultaneous administration of ABCB1-inhibiting CDKi with another cytotoxic agent that is an ABCB1 substrate might have synergistic antiproliferative effects. To test this hypothesis, we applied each of the three ABCB1 inhibitors that were most potent in our accumulation experiments (purvalanol A, roscovitine or olomoucine II) in combination with DNR (a commonly used anticancer drug and ABCB1 substrate) to several ABCB1-transduced or human tumor-derived cell lines. The CDKi-DNR combinations had significantly more pronounced synergistic effects on MDCKII-ABCB1 cells than on parental MDCKII cells. Thus, the synergistic effect of these combinations is clearly directly related to the expression of ABCB1. We postulate that CDKi increases the intracellular accumulation of DNR by inhibiting ABCB1, thus increasing its cytotoxic effect. Moreover, purvalanol A, olomoucine II and roscovitine also contribute to the cytostatic effect by their own cooperative proapoptotic activity.

In addition to genetically modified cells, two human carcinoma cell lines (HCT-8 and HepG2, derived from ileocecal adenocarcinoma and Caucasian hepatocyte carcinoma, respectively) were included in these studies since they abundantly express ABCB1 [24] and represent more clinically relevant settings than MDCK cells lines. We observed synergistic effects of CDKi and DNR combinations in both carcinoma cell lines, but weaker than those detected in MDCKII-ABCB1 cells, probably because expression of ABCB1 mRNA is an order of magnitude weaker in HCT-8 and HepG2 cells than in MDCKII-ABCB1 cells (Table 1). However, other factors such as the biotransformation of intracellularly accumulated DNR [43] or activities of other efflux transporters [44] may also affect the strength of the synergistic effects in various cells.

The synergistic activity of roscovitine, purvalanol A or olomoucine II in combination with DNR could offer a promising strategy in cancer treatment. There have been several reports on the synergistic effects of combinations of roscovitine with various cytotoxic agents, including paclitaxel [45], vinblastine, 5-fluorouracil and taxol [46] in vitro and others, e.g. doxorubicin, in vivo [47]. The synergistic activity is often attributed to reductions in survivin levels [45], [46], leading to increased induction of apoptosis. However, Appleyard et al. [47] observed no changes in p53 or survivin levels following combined applications of roscovitine and doxorubicin in a breast cancer xenograft model, suggesting that cell cycle arrest rather than apoptosis is the main mechanism of the enhanced antitumor effect. We provide here the first indications that the synergistic effect of DNR and CDKi might be at least partly due to interactive effects of the drugs on the ABCB1 transporter.

In conclusion, this is the first demonstration of the ability of five CDKi – purvalanol A, olomoucine II, roscovitine, flavopiridol, and SNS-032 – to inhibit ABCB1-mediated efflux, which can have a considerable impact on the pharmacokinetic behavior of simultaneously administered ABCB1 substrates. Identification of ABCB1 modulators is of great clinical interest, as these compounds are capable of reversing drug resistance and improving cancer chemotherapy [13]. The CDKi tested in our study act as such modulators and moreover contribute to a positive therapeutic outcome through their own cytotoxic activity. Thus, they have an advantage over “plain” ABCB1 modulators that only inhibit the efflux transporter without any other anticancer effect. As observed, administration of purvalanol A, olomoucine II, or roscovitine in combination with a cytostatic ABCB1 substrate has synergistic antiproliferative effects in ABCB1-expressing cells. Simultaneous administration of CDKi and ABCB1 substrates in the treatment of ABCB1-expressing tumors could, therefore, allow significant dose reductions of both concomitantly administered compounds (Table 2) and thus decrease their cumulative side effects and toxicity. We believe that our findings could be beneficial for further considerations of CDKi in pharmacotherapy, especially in cancer treatment as these compounds could have novel applications in circumventing multidrug resistance.
